# Time-resolved classification of dog brain signals reveals early processing of faces, species and emotion

**DOI:** 10.1038/s41598-020-76806-8

**Published:** 2020-11-16

**Authors:** Miiamaaria V. Kujala, Jukka-Pekka Kauppi, Heini Törnqvist, Liisa Helle, Outi Vainio, Jan Kujala, Lauri Parkkonen

**Affiliations:** 1 Department of Psychology, Faculty of Education and Psychology, University of Jyväskylä, PO Box 35, 40014 Jyväskylä, Finland; 2 Department of Equine and Small Animal Medicine, Faculty of Veterinary Medicine, University of Helsinki, PL 57, 00014 Helsinki, Finland; 3 Department of Neuroscience and Biomedical Engineering, Aalto University School of Science, P.O. Box 12200, 00076 Aalto, Finland; 4 Faculty of Information Technology, University of Jyväskylä, P.O. Box 35, 40014 Jyväskylä, Finland

**Keywords:** Behavioural methods, Electroencephalography - EEG, Electroencephalography - EEG, Neural decoding, Social evolution, Emotion, Perception, Neural decoding, Social behaviour, Animal behaviour, Animal physiology

## Abstract

Dogs process faces and emotional expressions much like humans, but the time windows important for face processing in dogs are largely unknown. By combining our non-invasive electroencephalography (EEG) protocol on dogs with machine-learning algorithms, we show category-specific dog brain responses to pictures of human and dog facial expressions, objects, and phase-scrambled faces. We trained a support vector machine classifier with spatiotemporal EEG data to discriminate between responses to pairs of images. The classification accuracy was highest for humans or dogs *vs.* scrambled images, with most informative time intervals of 100–140 ms and 240–280 ms. We also detected a response sensitive to threatening dog faces at 30–40 ms; generally, responses differentiating emotional expressions were found at 130–170 ms, and differentiation of faces from objects occurred at 120–130 ms. The cortical sources underlying the highest-amplitude EEG signals were localized to the dog visual cortex.

## Introduction

Domestic dogs (*Canis familiaris*) have developed a unique relationship with the human species over the last 15,000–30,000 years^1^, and they show remarkable social reactivity to their human packs. The dog coexistence with humans have even affected the formation of the dog facial musculature, enabling clearer facial expressions in communication with humans^2^. During the last decade, domestic dogs’ visual capabilities have proved to be better than previously expected, including their visual acuity^3^. Dogs also perform rather well in object recognition tasks. They are able to visually distinguish faces from non-faces, observing them spontaneously differently^4,5^ and they view faces in a holistic manner like humans, differentiating upright from inverted faces^6,7^. Dogs are also able to distinguish familiar faces from unfamiliar ones^7,8^ and they show both species-specific^9^ and emotion-specific^10–14^ differentiation of faces. Thus, face perception in dogs is relatively well characterized by behavioral responses and eye gaze tracking.

But how are faces processed in the dog nervous system? The basic face processing is similar to that of other mammal species, most studied in humans, monkeys and sheep^15,16^. Recent advances in utilizing non-invasive functional magnetic imaging (fMRI) in dogs have already revealed face specificity in the dog brain^17,18^. Furthermore, dog fMRI studies have shown differentiation in canine processing of human and dog faces^19^ and between different emotional expressions^20^. As in humans and other primates^21^, temporo–occipital brain regions appear important for processing of faces in dogs^17,18^. In dogs, the localization of face processing appears more variable than what is found in the face processing of humans^17,18^—possibly reflecting the differences in brain networks of different dog breeds^22^.

To date, the field has focused on the very basic functions of the dog visual processing; either on the behavioral distinction of socially relevant visual categories or addressing the focus of brain processing of faces with functional imaging, but the temporal aspects of face processing remain unclear. In humans, the dominant face-specific brain response peaks approximately 170 ms after the stimulus onset^23^. Emotional information may affect event-related potentials (ERPs) at different time windows depending on the context, modifying the early responses^24–26^ or the responses after 250 ms^27,28^. The first visuo-cortical ERPs of dogs have been detected at about 75–100 ms^29,30^, likely corresponding to the visual P1 and N1 components in humans at around 75–200 ms^31^. In our previous non-invasive electroencephalography (EEG) study, these early ERPs showed some reactivity to the content of the stimuli^30^. However, our previous study was more of a feasibility test and not optimized for comparing different categories of objects.

Here, utilizing non-invasive EEG deemed feasible in dogs^30,32^, we aimed to deepen the knowledge of the temporal dynamics of visual processing in dogs, related to different visual categories: faces, species, and emotion. We showed images of pleasant, threatening and neutral dog and human facial expressions as well as objects and phase-scrambled images to eight dogs that were previously trained with positive operant conditioning to undergo the EEG measurement procedures. We utilized a machine-learning approach in the analysis of the spatiotemporal EEG data to discriminate between brain responses to different image categories. Furthermore, we also employed a traditional analysis of event-related potentials to obtain a more comprehensive view of the possibilities of these two approaches in the analysis of canine ERPs. Moreover, to better connect our non-invasive EEG recordings with the existing literature on the brain areas underlying visual cognition, we also constructed the first EEG source model for dogs, and estimated the neural currents underlying the measured EEG signals.

Building on previous studies on processing of faces in the dog brain, we expected to be able to distinguish between brain responses to images containing faces *vs.* non-faces (objects and phase-scrambled images); images containing different species (dog *vs.* human); and images containing different emotional expressions. As aggressive or threatening faces yield strong behavioral responses in dogs in comparison with other expressions^11,12^, we expected especially threatening faces of both species to be distinguishable from neutral or pleasant faces.

## Results

### Event-related responses

**Figure 1:**
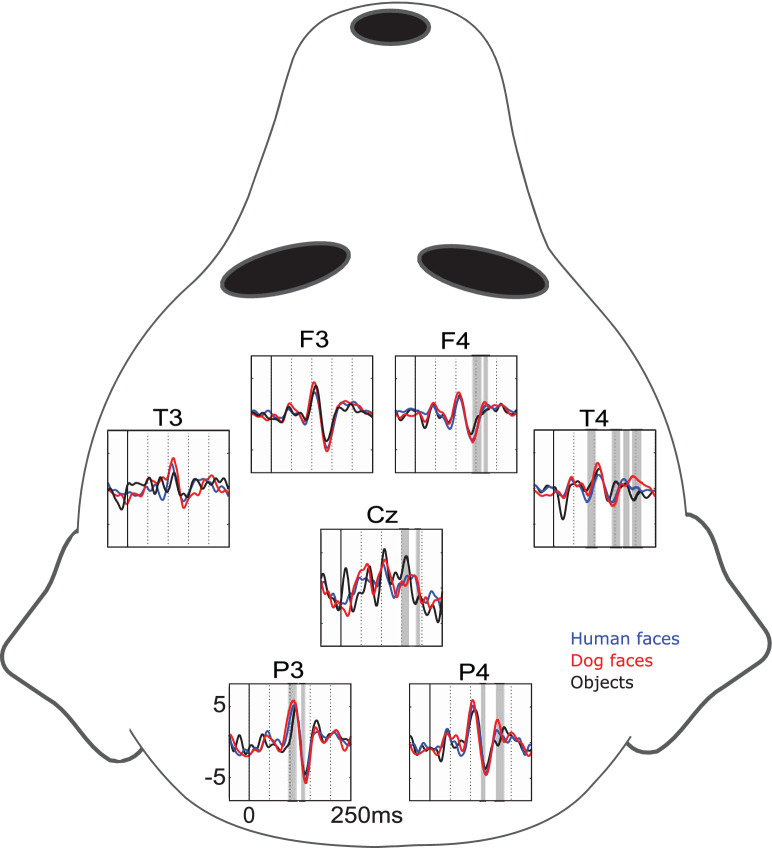
Grand-average evoked responses across the eight dogs for images of human faces (blue), dog faces (red) and objects (black) in the seven EEG channels (top = anterior; bottom = posterior). The schematic illustrates the electrode locations on the dog head. Grey dotted lines indicate 50 ms temporal intervals; solid grey bars indicate time windows where the responses to faces (human + dog) differed from objects.

Figure 1 shows the event-related responses—averaged across the eight dogs—to the images of humans, dogs and objects. The series of responses across the stimulus categories was markedly consistent, with highest-amplitude responses occurring at 105–110 ms after stimulus onset in the posterior P3 and P4 channels. A second notable response peaks at around 140 ms in the same channels. Both responses were prominent also in the frontal F3 and F4 channels.

The responses to face vs. object stimuli differed statistically at 5/7 channels (*p* < 0.05, paired-samples t tests). Face and object categories differed statistically significantly at the following channels and time windows, in the order of the onset latency of the time window: T4 at 80–104 ms, P3 at 92–119 ms, P3 at 123–139 ms, P4 at 123–139 ms, F4 at 139–166 ms, T4 at 139–166 ms, Cz at 146–170 ms, F4 at 162–182 ms, P4 162–182 ms, T4 at 166–189 ms, Cz at 182–197 ms and T4 at 186–213 ms.

Table 1 shows the statistically significant time windows obtained in the ANOVA analysis across the stimulus species (dogs, humans) and expressions (happy, aggressive, neutral). A main effect for species was seen in 3/7 channels between 45 and 100 ms, and in 1/7 channel at 201–205 ms. A main effect for facial expression was found in 4/7 channels: F4 at 29–37 ms; T3 at 127–135 ms; and P3 at 158–162 ms and 229–236 ms.

**Table 1:** ANOVA results testing the effects of species (sp), expression (exp) and interaction (int) on the amplitude of evoked responses.

Effect	Ch	Latency (ms)	*p* min	*p* max	HH	NH	AH	HD	ND	AD
exp	F4	29–37	0.014	0.039	− 0.038	0.146	− 0.308	0.183	0.434	− 1.199
sp	F4	45–100	0.003	0.035	− 0.515	− 0.756	− 0.932	0.123	0.462	0.117
sp	P4	68–80	0.005	0.023	− 1.057	− 0.160	− 0.646	1.087	1.068	0.109
sp	T4	76–88	0.012	0.029	− 1.273	− 0.530	− 1.434	− 0.533	0.482	0.507
exp	T3	127–135	0.027	0.049	− 1.094	0.758	− 0.654	− 1.866	2.040	− 0.427
exp	P3	158–162	0.011	0.027	2.386	− 1.659	0.377	1.227	− 0.940	0.050
sp	T4	201–205	0.035	0.038	0.465	− 0.645	0.045	1.619	1.470	0.927
int	F3	209–213	0.013	0.019	1.903	0.622	1.714	0.705	1.275	1.095
exp	P3	229–236	0.017	0.031	− 0.710	1.047	− 1.193	− 0.273	0.404	− 2.051

Subsequent significant time points are reported within the same temporal window, and the significant results are shown as average response amplitudes (µV) across the window. The results are listed in the ascending order according to the onset of the time window.

*HH* happy humans, *NH* neutral humans, *AH* aggressive humans, *HD* happy dogs, *ND* neutral dogs, *AD* aggressive dogs.

Table 2 shows planned contrasts (paired-samples t tests) that clarify the ANOVA facial expression and interaction effects within the significant time windows.

**Table 2:** The planned contrasts (paired-samples t tests) clarifying the ANOVA results (main effect of expression and interaction effect).

Ch	Latency	Value	HH versus NH	HH versus AH	NH versus AH	HD versus ND	HD versus AD	ND versus AD
F4	29–37	*p* min	0.659	0.416	0.267	0.582	0.096^^^	0.056^^^
		*p* max	0.715	0.489	0.329	0.799	0.166	0.066^^^
		T min	− 0.460	0.730	1.049	− 0.576	1.545	2.176
		T max	− 0.380	0.864	1.205	− 0.264	1.922	2.293
T3	127–135	*p* min	0.033*	0.289	0.089^^^	0.122	0.195	0.054^^^
		*p* max	0.174	0.974	0.150	0.293	0.481	0.263
		T min	− 2.650	− 1.148	1.618	− 1.759	− 1.431	1.217
		T max	− 1.513	− 0.033	1.974	− 1.137	− 0.744	2.311
P3	158–162	*p* min	0.119	0.123	0.131	0.022*	0.384	0.267
		*p* max	0.160	0.156	0.211	0.082^^^	0.444	0.385
		T min	1.571	1.591	− 1.713	2.032	0.810	− 1.205
		T max	1.777	1.752	− 1.377	2.921	0.930	− 0.926
P3	229–236	*p* min	0.070^^^	0.464	0.222	0.298	0.245	0.038*
		*p* max	0.286	0.761	0.260	0.608	0.267	0.103
		T min	− 2.140	0.316	1.224	− 1.124	1.207	1.875
		T max	− 1.155	0.775	1.341	− 0.537	1.270	2.551
F3	209–213	*p* min	0.038*	0.288	0.010*	0.102	0.172	0.460
		*p* max	0.040*	0.960	0.012*	0.235	0.335	0.854
		T min	2.516	− 0.052	− 3.484	− 1.880	− 1.522	0.190
		T max	2.545	1.149	− 3.370	− 1.298	− 1.035	0.781

The comparisons between happy, aggressive and neutral facial expressions within each time window are shown separately for human (HH, NH, AH) and dog (HD, ND, AD) faces.

^^^A trend towards significance; *significant at the level of *p* < 0.05.

### Machine-learning analysis

**Figure 2:**
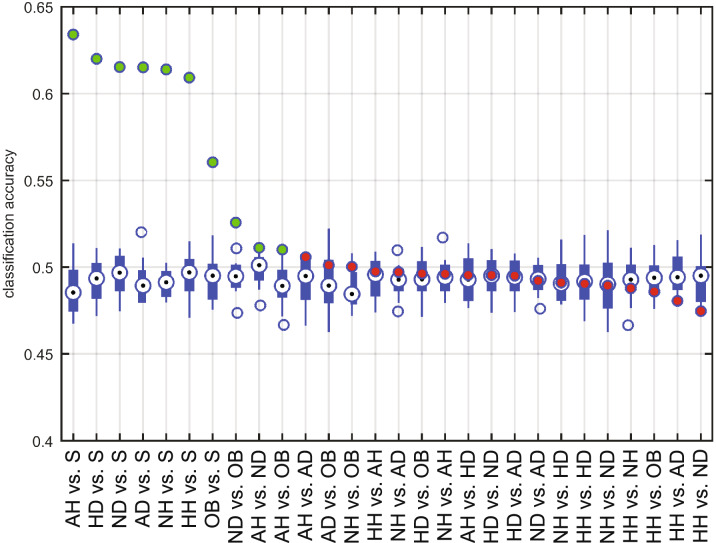
Average accuracy of each classification task. The tasks are ordered in the decreasing order of the accuracy: green circles indicate significant (*p* < 0.01) and red circles non-significant (*p* < 0.01) task accuracies. Blue box plots denote the null distribution obtained from the permutation test.

Figure 2 shows the average accuracy across the dogs for each binary classification task. Discrimination of any of the face stimulus categories from the scrambled images yielded the highest accuracies above 60% (range 60.9–63.4%; empirical chance level 48–52%). Comparable accuracies were obtained also with other classifiers (e.g., mean accuracy for face stimulus categories vs. scrambled images was > 60% with logistic regression using lasso regularization). Discrimination of objects from scrambled images resulted in the next highest average performance (56.1%), and the categories of neutral dogs (ND) and threatening/aggressive humans (AD) could be statistically significantly distinguished from objects (accuracy of 52.6% and 51.0%, respectively).

**Figure 3:**
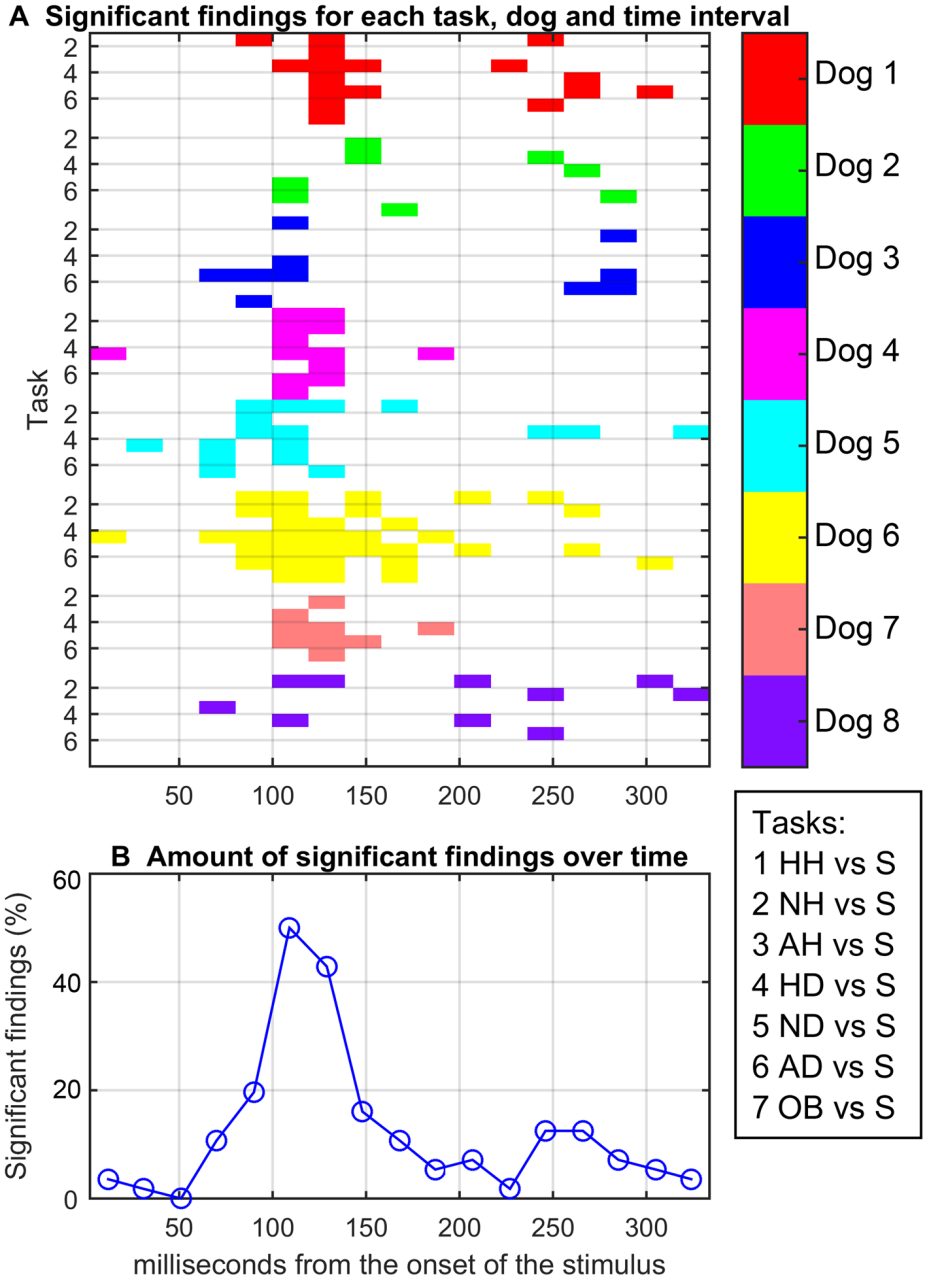
Time-resolved decoding of the scrambled versus other categories. (**A**) The matrix of statistically significant (*p* < 0.05, corrected) decoding results for each time interval (horizontal axis) and task pairs (vertical axis), separately for each dog. Colors indicate individual dogs, and each number (1–7) on the vertical axis corresponds to a different classification task (tasks are listed in the box at right). (**B**) The number of statistically significant classification results (%) over time, summarizing the findings across all dogs. Category abbreviations: *S* scrambled, *HH* happy human, *NH* neutral human, *AH* aggressive human, *HD* happy dog, *ND* neutral dog, *AD* aggressive dog, and *O* object.

Figure 3a shows a matrix of statistically significant classification results computed separately for distinct time intervals. Only the tasks discriminating scrambled images from the rest of the categories were selected for this analysis because the accuracy of these tasks was clearly better than random classification according to Fig. 2. Significant classifications were clustered around 100–140 ms after the onset of the stimuli. Moreover, there was another cluster around 240–280 ms. These two distinct time intervals can be seen clearly when significant classifications are summed across the tasks and dogs (see Fig. 3b): exactly half of the classifications (out of 56 corresponding to the 8 dogs and 7 tasks) were significant within the first peak (100–120 ms), and around 13% were significant around the later peak (240–280 ms).

**Figure 4:**
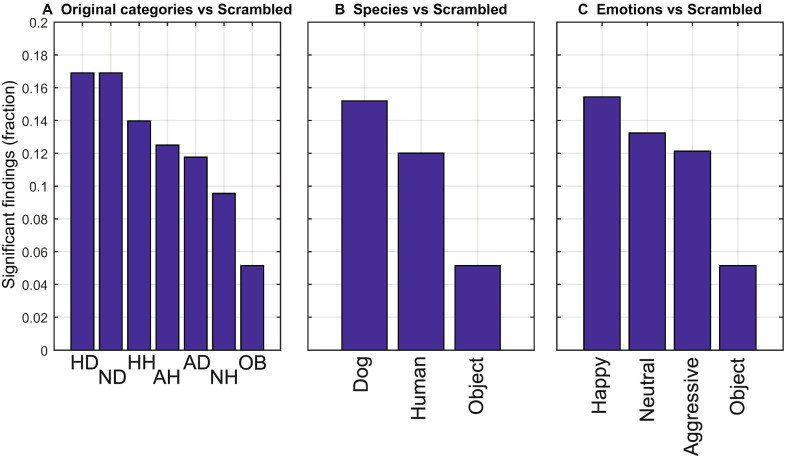
The fractions of statistically significant classification results across tasks, dogs and time points for three divisions of interest. (**A**) Species versus scrambled, (**B**) Expressions versus scrambled, and (**C**) Combined expressions and species versus scrambled. Within each division, discrimination results of the object category from the scrambled images are shown as a reference.

Figure 4 displays the fraction of statistically significant classification results across dogs, tasks, and time intervals (see the matrix of Fig. 3a) for the different tasks of interest based on three divisions (species, expressions, combined species and expressions). The division according to species (see Fig. 4a) reveals that the results of “dogs *vs.* scrambled” (15.2% of these tasks were significant) were more often significant than the results of “humans *vs.* scrambled” (12.0% of these tasks were significant). On the other hand, both humans and dogs were much more frequently discriminated from the scrambled images than from the images of objects (only 5.1% of these tasks were significant). When the division of the tasks is performed according to expressions (Fig. 4b), the task “happy *vs.* scrambled” was most frequently significant (15.4%), followed by “neutral *vs.* scrambled” (13.2%), and “aggressive *vs.* scrambled” (12.1%). Again, these relative numbers are higher compared to the reference task, object *vs*. scrambled.

Figure 4c shows the number of statistically significant classifications in terms of our original categorization consisting of both expression and species information. This division reveals that the tasks “HD *vs.* S” (16.9%) and “ND *vs.* S” (16.9%) yielded a higher number of statistically significant findings than the other tasks, including “HH *vs*. S” (13.9%), “AH *vs*. S” (12.5%), “AD *vs*. S” (11.8%), and “NH *vs*. S” (9.6%). Also in this case, the relative number of findings for the reference task, object *vs.* scrambled, was the lowest (5.1%) albeit the accuracy for these two tasks was very close to the chance level.

### Cortical sources

**Figure 5:**
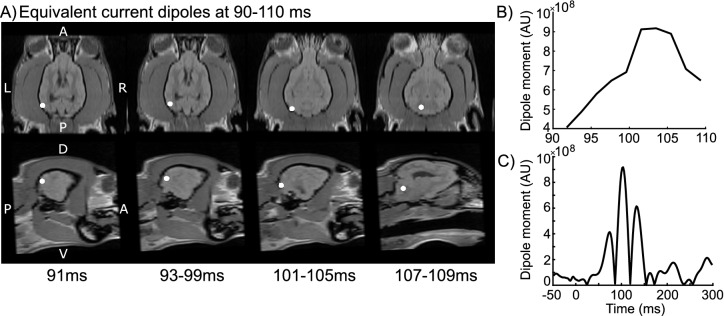
Cortical sources of the visual evoked response at 90–110 ms in one dog. (**A**) The location of ECDs with the highest goodness-of-fit in each time point (or time window, where the location remains constant) are shown with white circles, in both the axial (top row) and sagittal (bottom row) view of the dog brain. (**B**) Dipole moments of each of the ECDs fitted separately at each time point or time points with constant locations (91 ms, 93–99 ms, 101–105 ms and 107–109 ms). (**C**) The dipole moment of the ECD with highest amplitude between 90–110 ms shown over the whole epoch (from − 50 to 300 ms). *A* anterior, *P* posterior, *R* right, *L* left, *D* dorsal, *V* ventral.

Figure 5 shows the estimated cortical sources of the canine electrophysiological visual evoked responses. Modeling with equivalent current dipoles (ECD) revealed that the evoked responses with highest amplitude (at 90–110 ms) originated in the occipital cortex (Fig. 5a) as three distinct clusters of dipoles with goodness of fit of at least 93%. The largest dipole moments (of the 10 fitted dipoles) were observed for the cluster of dipoles fitted at 101–105 ms (Fig. 5b).

## Discussion

### Classification of faces and non-faces

Face perception is widespread across vertebrates^15,16^, thus investigation of facial processing in non-human species has two-fold implications. First, it adds to our understanding of the surrounding biological diversity and answers to our curiosity of the non-human minds—in this case, how does a dog see and process the social world? Second, it contributes to our views of the uniqueness of humans. How similar or different are we from our biological relatives, near or far in the evolutionary tree? Especially, here the question is: to what extent are the social cognitive processes, present in humans, shared across different species? Humans and dogs have had a special companionship dating back to prehistory^1^, and unraveling the emotional and face perception mechanisms in dogs shed light on these questions, also tackling the uniqueness of human brain processes.

In this work, our main achievements were: (1) We decoded the brain signals of individual dogs over the first 350 ms after image perception and predicted the stimulus category observed by the dog based on single event-related trials; (2) We clarified the important temporal windows characterizing dog event-related brain responses to processing emotional facial expressions; (3) We detected early responses approx. at 30–40 ms, that were highly pronounced for the threatening facial expressions, especially those of conspecifics; (4) We created an EEG forward model for one dog and estimated the cortical current generators of the most prominent visual brain responses. The decoding results highlight overlapping time windows in the brain processes of multiple dogs, showing the success of the method across individuals. Previous research with fMRI has utilized machine-learning approaches in the dog cognition research in detecting voxel activation patterns in listening to human words^33^; an fMRI preprint has also reported differentiation of human emotional expressions^20^.

As expected from the previous fMRI studies tapping specificity of faces in the canine brain^17–20^, we successfully discriminated the dog electrophysiological brain responses to both human and dog faces (happy/pleasant, neutral and aggressive/threatening) from the phase-scrambled non-face images. Highest classification accuracies were obtained for the tasks discriminating faces from scrambled images (accuracy in all task pairs above 60%). Statistically significant classification accuracies were also obtained for the task "objects *vs*. scrambled images" (56%). In the single-category comparisons where each emotion and species was independently compared without data pooling, brain responses to two categories of faces, neutral dogs and aggressive/threatening humans, could be separated statistically significantly from brain responses to objects. However, also the comparisons between other faces *vs.* objects were approaching significance.

When considering the number of SVM classifications (across time points and subjects) with all facial expressions of the same species pooled together, the percentage of correct classifications between dogs and scrambled images was the highest (15%), followed by the classification between humans and scrambled images (12%). Instead, the discrimination between objects and scrambled images was significant in only about 5% of the comparisons. Thus, the dog brain processing of dog faces differed most clearly from the scrambled images, highlighting the relevance of the conspecifics to dogs, previously shown behaviorally in eye gaze tracking^5^. The human faces also recruited clearly distinct brain patterns from the scrambled images, but to a lesser extent than dog faces.

Both behavioral and brain functional imaging studies have shown that dogs do differentiate between faces and non-faces^4,5,17,18^. In the current analysis of the event-related brain responses, face stimuli differed significantly from objects at approx. 80–200 ms from the stimulus onset. At the latencies of 123–213 ms, two or more channels picked up the face vs. object difference concurrently, adding to the reliability of the results. This time window both precedes and coincides with the face-specific response in humans around 170 ms^23^, but the current results suggest that the face processing in dogs may be more widespread both spatially and temporally than in humans. Importantly, the obtained differences are not likely to be due to the low-level stimulus properties of luminance or contrast, since the current face stimuli did not differ from the object stimuli in these metrics.

### Threat and emotional information of facial expressions

In previous behavioral and gaze-tracking experiments where dogs have viewed facial expressions from 2D presentations, aggressive or threatening faces have often provoked pronounced responses in dogs in comparison with other expressions^11,12,14,34^, and emotional effects have been present in fMRI data of dogs^19,20^. Therefore, we also expected brain responses especially to threatening expressions of both species to differ from the responses to neutral or pleasant expressions. Unfortunately, the comparisons between different emotional expressions of the same species did not reach sufficient classification accuracy in the machine-learning approach. Instead, in the conventional analysis, we detected an early response difference already at the latency of 30–40 ms, showing highest amplitude for the threatening/aggressive dog faces. The response latency suggests a subcortical, likely preconscious origin. This coincides with the studies of human amygdalar response to unconsciously detected, evident threat or conspecific fear^35^. Threatening stimuli are known to draw pre-attentive responses even in the absence of subjects’ conscious recollection of the stimulus^36^. In rodent studies, the early responsiveness of the amygdala has been characterized in the classical studies of conditioned fear^37^. Furthermore, our previous studies of dog eye gaze tracking clearly showed the behavioral response equivalent to amygdala-mediated “freezing response”—like failure to disengage from the threatening dog faces^12^. This phenomenon is well known in both human and non-human animals^38,39^.

The previous fMRI research has identified the face-responsive areas in the dog temporal cortex^17–19^, which has reciprocal connections with the basolateral amygdaloid complex^40^. In mammals, the lateral amygdalar nucleus is associated with fear conditioning, and the basolateral and basomedial nuclei are associated with anxiety and fear-related freezing^38^. Furthermore, amygdalar connections from the basal nucleus to the motor system have been detected in primates^41^ and cats ^42^*.* In monkeys, basolateral amygdala is highly reactive to threatening conspecific facial expressions^43^. These amygdala–neocortex connections may underlie our current findings of the early threat response in dogs. Canine temporal cortex bears some important similarities to the human brain network processing facial emotion: temporal cortex regions with stronger responses to dog faces have similar functional connectivity to the human superior temporal gyrus, and the areas with stronger responses to human faces have comparative functional connectivity to the human fusiform face area^19^.

As amygdala processes both negative and positive emotions^44^, we cannot rule out the effect of arousal in the current emotion-sensitive responses instead of the stimulus valence. Previously, when the current stimuli were rated by human subjects, threatening/aggressive dogs received the highest scores for both arousal and negative valence, followed by threatening/angry humans^45^. The early brain responses of dogs in this study follow the same pattern. Previously, also the dogs’ eye gaze separated both human and dog threatening expressions clearly from others, but in different ways: dog threat caused a sustained attention, but human threat caused an aversive response^12^. Both sustained attention^46^ and avoidance^47^ are well-known reactions to threat also in human studies. In the current study, we found early brain responses linked to threat detection, most pronounced for the threatening dog faces. Future work is needed to establish whether the different behavioral outcomes can be traced to differing stimulus properties (such as ecological validity and arousal) or observer properties (such as different anxiety profile of observers being represented in the sample).

In the emotional categories differentiated by machine-learning-based classification, threat was not easily detected. Brain responses to happy faces were generally the ones most often separated from the scrambled images: 17% of the SVM classification tasks were statistically significant between responses to happy dogs *vs.* scrambled images, and 14% were significant for distinguishing happy humans *vs.* scrambled images. Brain responses to neutral dogs were also as often separated (17%) from the scrambled images as happy dogs. The similar classification accuracy for happy and neutral dog faces may be partially due to similar conditions for classifications, as the dogs observed these expressions very similarly in our recent eye-tracking study^12^. Also in a recent fMRI study, happy human faces were the most prominently distinguished from other categories^20^. There may be several explaining factors for this, but the detected threat likely causes heightened vigilance, which may affect the signal-to-noise ratio of the data and consequently hinder the machine-learning analysis conducted on the basis of single trials.

Our event-related data analysis showed emotional expression-dependent effects at 127–170 ms from the stimulus onset, largely coinciding with the time window affected by emotional faces in comparable human ERP studies^26,27^. The time window is overlapping with the window differentiating faces from objects in the current study and detected partially in the same channels, suggesting that face processing in dogs may be connected to the processing of the affective content of the stimulus. In humans, the cortical face response is modulated by both attention and emotional expression, whereas the amygdala face response remains unaffected by attention^48^. In the current data the attention of dogs was non-manipulated, thus the biological relevance of the affective content of the stimuli likely affected the dogs’ attention and the face-sensitive cortical processing.

### Visual responses localized in the occipital cortex: distinction of species or low-level differences?

Dogs gaze dog and human faces partially differently^5^, and they have anatomically distinguishable brain activations for human and dog faces^19^. Here, we show differences between the dog event-related brain responses to human vs. dog faces at 45–100 ms, reflected in more than one EEG channels and replicating the finding from our previous study^30^ with a larger data set. Although early electrophysiological brain responses reflect differences in low-level visual properties within the stimuli, such as luminance or contrast^49,50^, we wanted to maintain the stimuli as natural as possible. Thus, the visual properties between stimulus categories were not equalized. For this reason, the dog faces differed from human faces both in their luminance and contrast, thus our results on the species-dependent effects within canine event-related responses cannot be distinguished from the effects of low-level visual properties.

In human electrophysiology research, interactive effects of low-level and socio-emotional properties have been reported already at the level of the early ERP components, around 100 ms from the stimulus onset^51^. However, the emotion-dependent effects in early ERP components are still under debate due to currently mixed findings^52^. It is possible that this early categorization of faces reflected in the dog brain responses is due to interactive effects of the low-level and biologically relevant stimulus properties, but unfortunately with the current data, we cannot conclusively resolve this issue for the dog event-related brain responses.

The brain responses at 45–100 ms, detected at the temporo-posterior channels, also had highest response amplitudes in the current study. The locations of the ECDs also indicate the source of these responses in the visual occipital cortices of the dog. This study is the first to estimate the cortical current sources of the non-invasively measured canine event-related brain potentials. To achieve this, we created the forward and inverse models of the dog head as the volume conductor, required to calculate the origins of the measured signals. Notably, the dog head has a thick musculature between the signal generation and the EEG measurement sites compared to a human head, affecting the signal conduction. Moreover, the limitations of the electrode network coverage have to be taken into account in this kind of studies. Nevertheless, our results give new directions and develop expectations for the future studies on canine cortical source localization.

### Time windows important for processing social visual information

As the time windows important for canine cognitive brain processes are largely unknown based on the previous literature, one of the main rationales of the present study was to clarify the temporal dynamics of brain processing of different visual categories in dogs. Previous studies have shown the first N1-like visual event-related responses of dogs to appear around 100 ms after stimulus onset^29,30^, and our previous study recording non-invasive ERPs suggested some category-dependent reactivity of the brain responses already at the early 100 ms responses^30^.

In the machine-learning approach of the present study, we clarified two time windows that yielded significant category-dependent classification of the brain responses. The first cluster of significant findings was at 100–140 ms and the second at 240–280 ms after the stimulus onset. Importantly, these processing windows were visible at the level of individual dogs. The first time window corresponds to the early 100 ms brain response already shown previously^30^ and this time window appears to be significant in differentiation of all other stimuli—faces and objects—from scrambled images. Notably in the second time window of 240–280 ms, none of the classification results arises between objects vs. scrambled images. This suggests that this time-window plays pronounced role in processing socially relevant information. This later time window roughly corresponds to the ERP latencies that have shown reactivity for emotional information in humans^27,28^. Additionally, we observed significant classification results in a number of time windows from the individual dogs, as shown in Fig. 3. These differences may be due to the anatomical differences between the individuals, but they may as well reflect the individual differences in the cognitive processing of the dogs. Currently, we have no way of obtaining the exact origins of these differences.

We conclude that the time windows for canine processing of visual social information obtained in the current study are much in line with the previous literature on human ERP literature, as well as the previous neuroimaging studies on dogs. The current results suggest a marked effect of the socio-emotional content to the dog brain responses, rendering the dog event-related responses comparable but more spatiotemporally distributed compared to the human responses.

## Methods

### Ethics statement

The study was performed in strict accordance with the Finnish Act on Animal Experimentation (62/2006), and all experimental protocols were approved by either the Viikki Campus Research Ethics Committee, University of Helsinki or the National Animal Experiment Board. All the experimental procedures related to the electroencephalography study had a prior approval by the Viikki Campus Research Ethics Committee, University of Helsinki (approval in the Board Meeting held on the 20th of March, 2013). At the time of the measurements, dogs were owned by the University of Helsinki, thus there was no need to obtain the informed consent from dog owners. No invasive procedures were applied in the EEG measurements, and only positive operant conditioning was used in the animal training. During the measurements, dogs were fully alert and conscious at all times with no medication, and measurements were conducted on voluntary basis: neither mechanical nor manual restraint was applied. The acquisition of magnetic resonance images (MRIs) had a prior approval by the National Animal Experiment Board (approval in the Board Meeting held on the 9th of November, 2011, #ESAVI/5794/04.10.03/2011).

### Subjects

Subjects were eight (8) healthy, neutered, purpose-bred beagles from five different litters. The dogs were raised as a social group and housed in a group kennel [6 males, 2 females, weighing 12.9. ± 1.9 kg (mean ± SD)], and all dogs were 6 years old at the time of the measurements. Purpose-bred dogs formed the subject group, since the aim was to avoid excess variation due to environmental effects. The subject dogs of the same breed, with comparable head sizes and forms, also enabled the comparison of the responses at a group-level. Furthermore, the dogs were already pre-trained for the task and had participated in similar non-invasive cognitive studies before^12,30,32^. They were keen to participate and performed the experiments well. All dogs were later re-homed to private families.

### Stimuli

Altogether, eight different categories of stimuli were presented. Stimuli were color photographs of dog faces with direct gaze (10 images of threatening/aggressive dogs (AD), 10 images of neutral dogs (ND), and 10 images as pleasant/happy dogs (HD); photographs of human faces with direct gaze (10 threatening/aggressive humans (AH), 10 neutral humans (NH), and 10 pleasant/happy humans (HH); 10 images of general household objects (OB) and 10 images of abstract pixel compositions, phase-scrambled from the neutral dog faces (S). The stimulus images were acquired from our previous studies with further details of the human and dog faces^12,45^.

Differences in low-level visual properties of the stimulus categories could spuriously contribute to classification, thus these properties were calculated for each category. Luminance of the faces/objects were the following (mean ± SD): AD 112 ± 25; ND 103 ± 33; HD 120 ± 42; AH 135 ± 19; NH 140 ± 24; HH 142 ± 23; OB 130 ± 21. The RMS contrasts were the following (mean ± SD): AD 15.8 ± 0.2; ND 15.4 ± 0.7; HD 15.8 ± 0.3; AH 15.9 ± 0.1; NH 15.8 ± 0.2; HH 15.9 ± 0.1; OB 16.0 ± 0.0. The face stimuli (AD, ND, HD, AH, NH, HH) did not differ statistically from the objects (OB) in either luminance or contrast, but human faces differed from dog faces in both luminance (*p* = 0.002; two-sample t test) and contrast (*p* = 0.04; two-sample t test). The different emotional expression categories did not differ from each other (AD + AH vs. HD + HH; AD + AH vs. ND + NH; ND + NH vs. HD + HH) in either luminance or contrast.

### Preprocessing

To reduce the effect of muscular and other artefacts in the data, independent component analysis (ICA) was applied^53^, separately to each measurement block. ICA decomposition was performed for the time window from one second before the first and two seconds after the last stimulus onset within the block. The artefactual components within each block were identified based on visual inspection of the topography and spectral content of the components. The influence of the components that were labeled artefactual was removed by including only the other components in the reconstructed EEG data. In addition to the ICA, the possible electric leakage of the stimulus trigger signal to EEG channels was removed with a general linear model.

### Stimulus presentation

Stimulus images were displayed on a standard 22″ LCD monitor and were approx. 14.6 × 16.0 cm^2^ (width × height) in size, overlaid on a gray background screen of 47.4 × 29.7 cm2 (1680 × 1050 pixels), and presented at a frame rate of 60 Hz. Stimulus presentation was controlled with Presentation software (https://nbs.neuro-bs.com/) running on a standard PC.

Stimuli were presented in a pseudorandomized order at a distance of 70 cm, while the dogs laid still on a 10-cm thick Styrofoam mattress and leaned their jaw on a purpose-designed u-shaped chin rest, as in our previous studies^5,30^. Each stimulus was shown for 500 ms with a uniformly-distributed random inter-stimulus-interval of 720–1560 ms, within 5 separate stimulus blocks of 15–20 stimuli per block; altogether, 85–88 stimuli were shown during one measurement session (10–11 stimuli per category). Each block of the session started with a stimulus from a different category, and the total duration of each measurement session was approx. 2 min 30 s. Between stimulus blocks, the dog was rewarded with a piece of food and let to settle again on the measurement mattress. The total measuring time within one measurement session, including rewarding periods, ranged from 5 to 20 min depending on the re-settling needed by the dog; only one session was recorded per day per dog. The data were gathered in fourteen separate recording sessions, each on a separate day, one week between the measurement days.

### Data acquisition

The measurements took place at the facilities of Faculty of Veterinary Medicine, University of Helsinki. Dogs were pre-trained, with operant-positive conditioning (clicker), for the non-invasive EEG task in our previous studies^30,32^. During the data acquisition, they wore neonatal EEG electrodes (Unilect 40555 with bio-adhesive solid gel, 22 × 22 mm^2^, Unomedical a/s, Denmark) designed for newborn babies and a dog vest carrying the portable EEG amplifier (weighing 200 g). They settled in the measurement mattress on their own and rested their heads at a chin rest but were not restrained in any way.

To attach the electrodes, hair from the top of the dog's head was shaved and the skin was cleaned with isopropyl alcohol to ensure a proper contact of the electrodes with the skin. Subsequently, drops of cyanoacrylate were applied to the edges of the electrode pads, and medical skin tape was applied on top of the electrodes to ensure their attachment.

The EEG data were acquired with an ambulatory Embla Titanium-recorder and RemLogic 2.0—software (Embla Systems, Colorado, USA). The EEG setup comprised 7 electrodes: F3 and F4 laterally approximately over the left and right frontal cortex, respectively; T3 and T4 over the lateral temporal cortices; P3 and P4 over the parieto-occipital cortices, and Cz in the middle; reference electrode was placed on the right ear and a ground electrode in the lower back. The EEG signals were band-pass filtered to 0.15–220 Hz and digitized at 512 Hz.

### Conventional sensor-level analysis

For the analysis of event-related responses, the data were first band-pass filtered to 2–40 Hz. Trials that showed amplitude deviations of more than 100 μV within the time window of interest (− 150 to 350 ms with respect to the stimulus onset) were rejected. As a result, 114–146 trials were included in the subsequent analyses per condition in individual dogs; in total, 1043–1078 trials per condition were acquired. Evoked responses were then averaged within each dog and experimental condition. The average signal in the baseline window (− 150 to 0 ms) was subtracted from the responses.

Effects of the stimulus categories were tested with analysis of variance (ANOVA) across the stimulus species (dogs, humans) and expressions (happy, aggressive, neutral). In this hypothesis-driven analysis, the responses were tested statistically with overlapping, 16-ms long time windows moving in steps of 4 ms, in the range of 0 to 250 ms with respect to stimulus onset. The threshold for significant findings was set at *p* < 0.05, uncorrected for the number of time-windows but only findings of ≥ 2 contiguous significant time windows are reported. Additionally, the differences between event-related responses to face vs. object stimuli were tested separately as a planned contrast, with similar procedures as above.

### Analysis with machine learning

We trained a support vector machine (SVM) classifier^54^ from spatiotemporal EEG data, band-pass filtered to 2–25 Hz, to discriminate between epochs originating from different image categories. For the machine-learning analysis, all recorded trials (epochs) were included to ensure the data suffiency, yielding 128–155 trials across the eight conditions and dogs. Before classifier training, we averaged temporally adjacent data points using a non-overlapping time window of 20 ms. This way the dimensionality of the original data was reduced with only a minimal loss of temporal information. After vectorization of the resulting time–channel matrix, we obtained 301-dimensional feature vectors for classifier training and testing. We trained a separate SVM classifier for each binary classification task, leading to (7·8)/2 = 28 classifiers for each dog, using the statistics and machine learning toolbox of Matlab (MathWorks, Inc., Natick, MA, USA).

We used a linear kernel in the SVM and applied tenfold cross-validation to estimate the test accuracy of the trained classifiers. We also constructed classifiers for each 20-ms time interval separately to investigate temporal evolution of discriminative information between categories from the onset of the stimulus. In this case, the dimension of the feature vectors was 7, corresponding to the number of EEG channels. For each classifier, we conducted a permutation test to assess whether the classification accuracies were significantly above the chance level^55^. For this purpose, we randomly permuted category labels of the training data 200 times prior to classifier training and testing and generated a null distribution from the corresponding classification accuracies. The significance threshold corresponding to *p* < 0.05 was obtained using a maximum-statistics approach^56^ simultaneously across all time-windows and category-pairs. This significance threshold was also Bonferroni-corrected for the number of dogs.

### Source modeling of evoked responses

The cortical sources of the evoked responses were estimated in one dog whose anatomical head MR images could be obtained. Here, equivalent current dipole (ECD) modeling was applied^57^, implemented in the FieldTrip toolbox^58^.

The structural T1-weighted head MRI was acquired with 0.2-T open magnet (Esaote S.p.A, Genova, Italy) at the Veterinary Teaching Hospital, University of Helsinki, using a spoiled-gradient echo sequence with a matrix size of 256 × 256, time of repetition 1520 ms, and field of view 180 × 190 mm^2^. In total, 41 slices were acquired with a thickness of 2.5 mm and a 0.2-mm gap between slices. During the data acquisition, the dog was sedated and resting in the MR scanner.

The MRIs were segmented using Freesurfer version 5.3^59^, and a boundary element method-based EEG forward model consisting of the brain, skull and scalp compartments with conductivities of 0.3 S/m; 0.006 S/m and 0.3 S/m, respectively, was constructed with MNE-Python^60^. Possible source locations were considered in a regular volumetric grid with 2-mm spacing between the source points.

Prior to source estimation, the EEG data were band-pass filtered to 2–25 Hz and the evoked responses across all facial stimulus categories were averaged using a rejection threshold of 50 μV, resulting in 719 epochs. The cortical sources of the most prominent visual EEG response in the time window of 90–110 ms were estimated by fitting an equivalent current dipole (ECD) separately at each time point within the 90–110-ms window. In addition to the dipole location and orientation, dipole strength and goodness-of-fit were estimated separately at each time point.

## Data Availability

The datasets recorded and analyzed in the current study are available from zenodo.org with the following digital object identifier: https://doi.org/10.5281/zenodo.4114599.
