# Publisher Correction: Time-resolved classification of dog brain signals reveals early processing of faces, species and emotion

**DOI:** 10.1038/s41598-021-85718-0

**Published:** 2021-03-19

**Authors:** Miiamaaria V. Kujala, Jukka‑Pekka Kauppi, Heini Törnqvist, Liisa Helle, Outi Vainio, Jan Kujala, Lauri Parkkonen

**Affiliations:** 1 Department of Psychology, Faculty of Education and Psychology, University of Jyväskylä, PO Box 35, 40014 Jyväskylä, Finland; 2 Department of Equine and Small Animal Medicine, Faculty of Veterinary Medicine, University of Helsinki, PL 57, 00014 Helsinki, Finland; 3 Department of Neuroscience and Biomedical Engineering, Aalto University School of Science, P.O. Box 12200, 00076 Aalto, Finland; 4 Faculty of Information Technology, University of Jyväskylä, P.O. Box 35, 40014 Jyväskylä, Finland

Correction to: *Scientific Reports* 10.1038/s41598-020-76806-8, published online 16 November 2020

The original version of this Article contained typographical errors.

In the Introduction section,

“In humans, the dominant face-specific brain response peaks approximately 170 ms after the stimulus onset for review,^23^.”

now reads:

“In humans, the dominant face-specific brain response peaks approximately 170 ms after the stimulus onset^23^.”

In the Discussion section, under the heading ‘Classification of faces and non-faces’,

“This time window both precedes and coincides with the face-specific response in humans around 170 ms for review, see^23^.”

now reads:

“This time window both precedes and coincides with the face-specific response in humans around 170 ms^23^.”

Also in the Discussion section, under the heading ‘Threat and emotional information of facial expressions’,

“In rodent studies, the early responsiveness of the amygdala has been characterized in the classical studies of conditioned fear for review, see^37^.”

now reads:

“In rodent studies, the early responsiveness of the amygdala has been characterized in the classical studies of conditioned fear^37^.”

And,

“This phenomenon is well known in both human and non-human animals for reviews, see^38,39^.”

now reads:

“This phenomenon is well known in both human and non-human animals^38,39^.”

And,

“In mammals, the lateral amygdala-nucleus is associated with fear conditioning, and the basolateral and basomedial nuclei are associated with anxiety and fear-related freezing for review, see^38^.”

now reads:

“In mammals, the lateral amygdala-nucleus is associated with fear conditioning, and the basolateral and basomedial nuclei are associated with anxiety and fear-related freezing^38^.”

These errors have now been corrected in the PDF and HTML versions of the Article.

